# Outcomes of Burn Patients Admitted Initially to a Non-Burn Center Intensive Care Unit in Romania: A Retrospective Exploratory Study

**DOI:** 10.3390/medicina62030443

**Published:** 2026-02-26

**Authors:** Olga Grăjdieru, Constantin Bodolea, Vlad Moisoiu, Cristina Petrișor, Bogdan Tigu, Andrea Szekely, Rareș Streza, Cătălin Constantinescu

**Affiliations:** 1 Department of Anesthesia and Intensive Care, Iuliu Hatieganu University of Medicine and Pharmacy, 400012 Cluj-Napoca, Romania; olgadoinag@yahoo.com (O.G.); constantin.bodolea@umfcluj.ro (C.B.); petrisor.cristina@umfcluj.ro (C.P.); rares.streza@yahoo.com (R.S.); 2 Intensive Care Unit, Emergency Clinical Hospital, 400006 Cluj-Napoca, Romania; 3 Municipal Clinical Hospital, 400139 Cluj-Napoca, Romania; 4 MedFUTURE Research Center for Advanced Medicine, Iuliu Hatieganu University of Medicine and Pharmacy, 400349 Cluj-Napoca, Romania; vlad.moisoiu@gmail.com (V.M.); adrianbogdantigu@gmail.com (B.T.); 5 Department of Anaesthesiology and Intensive Therapy, Semmelweis University, 1085 Budapest, Hungary; szekelyandi64@gmail.com

**Keywords:** burn injuries, intensive care unit, six-month survival, non-burn center, retrospective cohort study, multidisciplinary care, Romania

## Abstract

*Background and Objectives*: Many burn patients are initially admitted to non-burn center intensive care units (ICUs) due to resource constraints, geographic barriers, or delayed transfer. Their management requires multidisciplinary, phase-specific clinical and assistive practices, yet outcomes from non-specialized centers remain scarce. This study aimed to (1) determine six-month survival among all adult burn patients initially admitted to a non-burn center ICU, (2) identify clinical predictors of mortality, and (3) explore reasons for transfer to burn centers within a resource-limited healthcare setting. *Materials and Methods*: We conducted a retrospective observational cohort study including 42 adult burn patients initially admitted to the ICU of a regional non-burn hospital in Romania (2019–2024). Of these, 24 were treated entirely in the non-burn center (initially treated in the ICU and then further managed on the same hospital’s wards), and 18 were, after initial ICU stabilization, subsequently transferred to burn centers. Six-month survival was assessed using Kaplan–Meier analysis. Logistic regression and Cox proportional hazards models were used to assess associations with mortality. Clinical trajectories and transfer decisions were also analyzed. *Results*: Six-month survival was 61.9% (95% CI 48.8–78.5), with most deaths occurring within 60 days. Total body surface area (TBSA) (HR 1.05, 95% CI 1.02–1.08, *p* < 0.001) and acute kidney injury (AKI) (HR 3.48, 95% CI 1.18–10.29, *p* = 0.024) were independently associated with mortality. Patients transferred to burn centers had greater TBSA (median 35% vs. 15%, *p* = 0.003), consistent with severity-driven referral decisions. *Conclusions*: Among all burn patients initially admitted to a non-burn center ICU, six-month survival was 62%, with TBSA and AKI independently associated with mortality in adjusted analyses. These findings underscore the importance of phase-specific, multidisciplinary care pathways, including early resuscitation, renal and hemodynamic monitoring, coordinated nursing care, rehabilitation planning, and transfer protocols to improve outcomes.

## 1. Introduction

The number of burn victims who survive the initial injury and reach the hospital has increased due to improved access to high-quality care and multidisciplinary teams [1]. However, not all hospitals are equipped to manage such complex cases, and critically ill burned patients often develop multiorgan failure, making it impossible to transport them to a specialized burn center [2]. Consequently, these patients sometimes have to be treated in resource-limited environments [3,4]. Data from high-income nations where regionalized burn care is established report that between 57% and 71% of major burn patients are treated in burn centers [5,6], but this is not the case across all healthcare systems worldwide. In countries outside the United States, national reports indicate that a proportion less than 60% of hospitalized burn patients are admitted to specialized burn centers [7].

In Romania, only a limited number of hospitals provide care for major burn patients. Due to the shortage of specialized beds at the national level, some patients remain for their entire course of care in regional hospitals, and sometimes they need to be transferred to hospitals abroad. There is evidence that admission to a specialized burn center is associated with a significant survival benefit compared to a general critical care unit [8,9]. Moreover, regionalization has been shown to improve survival compared to non-burn hospitals, which exhibit variability in the capacity and quality of care, and where about a third of burn patients are still being treated [6,10].

Burns can result in persistently high morbidity and mortality rates in the long term [11]. The overall survival of these patients depends on the mechanism of injury, the quality of initial resuscitation care, and the development of sepsis and multiorgan failure [12]. A European data analysis of 186,500 burn patients from different countries reports a decrease in both incidence and mortality, attributed to improved situational awareness and better access to treatment [13]. However, available information estimates that burn injuries affect hundreds of thousands of individuals annually, though the exact incidence remains uncertain [14,15]. Moreover, the apparent seasonal variation in burn occurrences has yet to be clearly characterized [16]. Still, there is significant heterogeneity among studies, and worldwide data on burn patients are not always up to date. Establishing national registries and systematic data gathering would provide a more accurate representation of the burn injury burden within each country.

The primary objective of this study was to report the overall survival of all adult burn patients admitted initially to an intensive care unit (ICU) of a non-burn center, regardless of whether they were later transferred to a burn center or treated locally for their entire course of care, using Kaplan–Meier survival analysis to describe 6-month survival outcomes. The secondary objectives were to identify clinical predictors of mortality through univariate and multivariate analyses, including the Cox proportional hazards model to determine independent prognostic factors, and to explore the main reasons for transfer of burn patients to burn centers, providing insight into local referral practices in a resource-constrained healthcare setting.

Integrating these results and pitfalls into a contemporary multidisciplinary approach to burn management emphasizes the necessity of complex coordinated teams overseeing acute stabilization, wound care, grafting, and rehabilitation for long-term recovery, resources that are often limited in non-burn settings. These findings could help enhance the quality of care for burn patients in non-burn centers and especially in emerging healthcare systems. Currently, while extensive research on the management of burn patients exists, comparative studies or large-scale randomized trials evaluating outcomes in specialized versus non-specialized settings remain scarce. This lack of information underscores the need for further investigation to determine how improvements in critical care settings influence survival, reduce complication rates, optimize care, and identify key factors responsible for better outcomes.

## 2. Materials and Methods

### 2.1. Study Design and Participants

We conducted a retrospective, observational cohort study in which we included all adult burn patients initially admitted to the ICU of a regional emergency hospital in Cluj-Napoca, Romania, over a six-year period (1 January 2019 to 31 December 2024). This hospital provides comprehensive medical care, but it is not designated as a specialized burn treatment center. Data were collected from the hospital’s electronic database and from the observation sheets that were made available. Inclusion criteria were age ≥ 18 years, admission to the ICU in the 2019–2024 period, having the primary diagnosis of burn injuries (based on the International Classification of Diseases, 10th Revision, codes T20-T32), and availability of data on six-month survival status. Patients with burn injuries who did not reach the ICU (died in the pre-hospital setting) and those with non-burn-related primary diagnoses were not included. No patients were excluded due to missing data.

To minimize selection bias, all consecutive eligible patients were included without sampling. The study size was determined by including the entire available cohort over the six-year period, with no formal sample size calculation performed due to the exploratory nature. Due to its retrospective study design, potential selection and information biases could not be fully excluded, but they were addressed by cross-verifying data from electronic files and observation sheets and using standardized diagnostic criteria for key variables.

The transfer to specialized burn care services was made in accordance with Romanian national burn referral guidelines, which include thresholds based on TBSA (>20% for adults), the presence of inhalation injury and other special areas, and severity scores such as the Baux Score. Follow-up six-month survival was ascertained through linkage with the Cluj County Directorate for Population Records, which provided dates of death.

The gathered information was anonymized to keep the confidentiality of the patients. This study was conducted and reported in accordance with the RECORD (Reporting of Studies Conducted Using Observational Routinely Collected Data) guidelines [17] and the STROBE guidelines for observational studies (Supplementary File). Ethical approval was obtained from the Ethics Committee of the Emergency County Clinical Hospital and the University of Medicine and Pharmacy “Iuliu Hațieganu” Cluj-Napoca (No. 197/7 July 2025). The requirement for informed consent was waived by the ethics committee due to the retrospective nature of the study and the use of anonymized data.

### 2.2. Objectives and Variable Definitions

The primary objective of this study was to describe six-month survival among all adult burn patients initially admitted to the ICU of a non-burn center, regardless of subsequent transfer to a specialized burn center or continued local treatment, using Kaplan–Meier methods. Secondary objectives were to identify independent predictors of mortality through univariate and multivariate modeling, including the Cox proportional hazards model, and to explore reasons for transfer to burn centers, providing insight into local referral practices in a resource-constrained healthcare setting.

Mortality (primary outcome) was defined as all-cause death within 180 days of ICU admission. Patients alive at six months were censored at 180 days if their follow-up exceeded this period. There was no loss to follow-up due to the administrative linkage.

We analyzed other variables such as total body surface area (TBSA—continuous, as a percentage, estimated using the Lund–Browder chart at initial assessment), mechanism of burn injury, emergency room length of stay, ICU length of stay, hospital length of stay, demographic factors (age, gender), and organ function at ICU admission (assessed via standard laboratory and clinical parameters). To assess the impact of associated disease on the main outcome, we used the Charlson Comorbidity Index (CCI), which can estimate the 10-year survival based on the calculation of age and different comorbidities [18]. We also evaluated complications developed during admission (e.g., sepsis was diagnosed according to the Surviving Sepsis Campaign guidelines [19]; renal dysfunction and acute kidney injury (AKI) were identified based on KDIGO criteria [20,21]; coagulopathy was identified by using abnormal values of international normalized ratio (INR) and activated partial thromboplastin time (APTT) together with disseminated intravascular coagulopathy (DIC) score [22,23]). Based on its high sensitivity and specificity values in burn patients, the Revised Baux (R-Baux) Score was used, and it was calculated as (TBSA + Age + [17*R]), where R is 1—inhalation injury present, confirmed via bronchoscopy, or 0—inhalation injury absent [24].

### 2.3. Data Analysis

Statistical analyses were performed using R (version 4.4.3; R Foundation for Statistical Computing, Vienna, Austria) run in RStudio (Posit Software, Boston, MA, USA). Categorical variables were summarized as frequencies and percentages. Chi-square or Fisher’s test (when count < 5) was used to analyze contingency tables for categorical tables. The normality of distribution was assessed using the Shapiro–Wilk test and histogram visualization. For normally distributed data, the *t*-test was applied, which was reported as mean (SD), and for non-normally distributed data, the Wilcoxon rank-sum test was used, reported as median (IQR). Survival was analyzed as time-to-event, using Kaplan–Meier survival analysis, with log-rank tests. Univariate analyses were performed to describe associations of variables with mortality. Because only 16 events occurred, the multivariate models were restricted to only two predictors to reduce events-per-variable overfitting. Multivariable logistic regression was used to model the probability of death by 180 days (odds ratios (ORs) and 95% CI), while the Cox proportional hazards model assessed time-to-death within 180 days (hazard ratios (HRs) and 95% CI), because follow-up time and event timing were available for all patients. Variable selection for the multivariate model was based on expertise, univariate results, best fit, and avoidance of multicollinearity. No subgroup analyses were examined. For the variables analyzed, there were no missing values. A *p*-value under 0.05 was considered statistically significant.

## 3. Results

### 3.1. Study Patients

Based on the inclusion criteria, this retrospective cohort included 42 burned patients admitted initially to the ICU of the Emergency County Clinical Hospital in Cluj-Napoca, Romania, over a six-year period (2019–2024). The cohort comprised 24 patients (57%) treated for the entire course of care in the non-burn center, transitioning from ICU to the same hospital’s wards for continued care, and 18 (43%) patients transferred to a specialized burn center after a period of initial management in the non-burn center ICU. The mean age was 57.0 years (SD 18), with male preponderance (74%). The overall median TBSA burned was 20% (IQR 27), and the median CCI was 2 (IQR 4). Patient characteristics are outlined in Table 1.

### 3.2. Overall Survival

**Figure 1:**
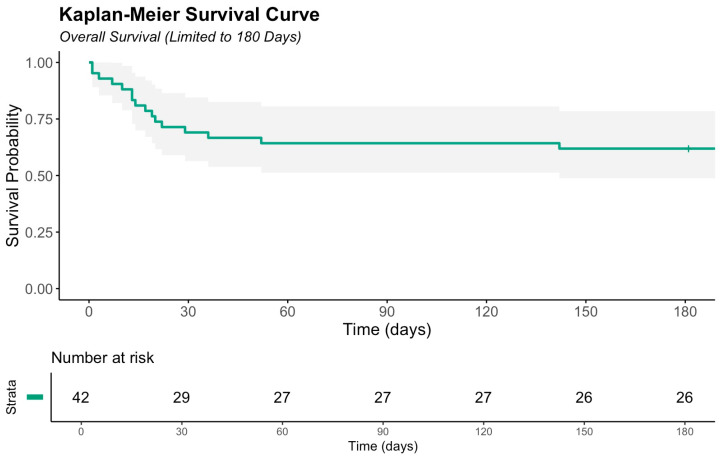
Kaplan–Meier overall survival curve of the study cohort (N = 42). The solid line represents the Kaplan–Meier survival curve for overall survival up to 180 days. The shaded gray area denotes the 95% confidence interval. Tick marks indicate censored observations. The number at risk at each time point is shown below the x-axis.

During the 180-day follow-up, 16 deaths (38.1%) occurred among the 42 patients in the cohort. The estimated 6-month overall survival was 61.9% (95% CI 48.8–78.5) (Figure 1). The majority of deaths occurred within the first 60 days after injury, followed by a plateau. At 180 days, 26 patients remained alive and at risk.

**Figure 2:**
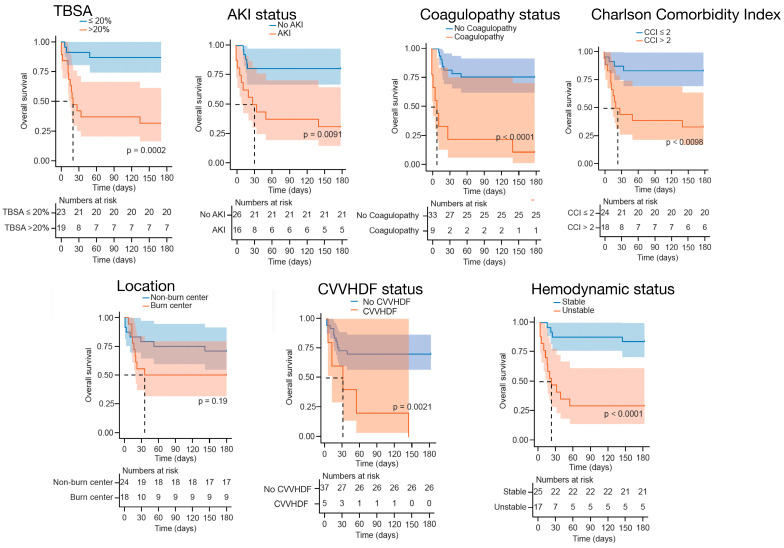
Kaplan–Meier survival estimates according to major clinical variables. Survival probabilities up to 180 days are shown by TBSA, AKI, Charlson Comorbidity Index, hemodynamic status, CVVHDF use, coagulopathy, and treatment location. Shaded areas represent 95% confidence intervals.

Kaplan–Meier survival curves at 180 days demonstrated significant differences in survival across several clinical factors (Figure 2). Patients with larger burn extent (TBSA > 20%) showed markedly lower survival compared with those with ≤20% TBSA (6-month survival 31.6% (95% CI 16.3–61.2) vs. 86.9% (95% CI 74.2–100), *p* = 0.0002). Most deaths among patients with larger burns occurred within the first two months after injury. The presence of AKI was also associated with reduced survival (31.2% (95% CI 15.1–64.6) vs. 80.8% (95% CI 66.9–97.4), *p* = 0.0009). Similarly, patients with a higher CCI (>2) had significantly poorer outcomes than those with CCI ≤ 2 (33.3% (95% CI 17.3–64.1) vs. 83.3% (95% CI 69.7–99.7), *p* = 0.001). Hemodynamic instability was a strong predictor of mortality, with survival of 29.4% (95% CI 14.1–61.4) compared with 84.0% (95% CI 70.8–99.7) among stable patients (*p* < 0.001). Those requiring CVVHDF had substantially lower survival (0% vs. 70.3% (95% CI 56.9–86.7), *p* = 0.002). The presence of coagulopathy was associated with the most profound impact on outcome, with survival of only 11.1% (95% CI 1.8–70.5) compared to 75.8% (95% CI 62.5–91.9) in patients without coagulopathy (*p* < 0.0001).

Although there was no statistical difference regarding treatment location (*p* = 0.20), where the estimated survival was 70.8% (95% CI 54.8–91.6) for patients treated in the non-burn hospital and 50.0% (95% CI 31.5–79.4) for those transferred to a burn center, it is worth mentioning that among patients who survived, the median TBSA was higher in those treated in the burn centers (TBSA 35% [IQR 24] vs. 10% [IQR 10], *p* = 0.0014).

### 3.3. Univariate Analysis

In univariate logistic regression analysis (Table 2), several clinical and physiological variables were analyzed among adult burn patients.

Increasing age and TBSA burned were associated with higher odds of death; for each additional year of age, the odds of mortality increased by approximately 6% (OR = 1.06, 95% CI 1.02–1.12, *p* = 0.007), and for each 1% increase in TBSA, the odds increased by 8% (OR = 1.08, 95% CI 1.03–1.13, *p* = 0.002). A higher CCI score (>2) was also strongly associated with mortality (OR = 10.0, 95% CI 2.52–48.1, *p* = 0.002).

Among complications, the presence of AKI (OR = 9.24, 95% CI 2.34–43.1, *p* = 0.002) and coagulopathy (OR = 25.0, 95% CI 3.79–502.4, *p* = 0.005) were both significantly associated with higher odds of death. Hemodynamic instability on admission was another strong predictor, increasing mortality odds by more than twelvefold (OR = 12.0, 95% CI 2.8–68.6, *p* < 0.001). Patients requiring CVVHDF also had markedly higher mortality risk (OR = 25.3, 95% CI 2.54–3432.9, *p* = 0.003, Firth’s correction applied).

In contrast, treatment location (transferred to a burn center vs. treated locally), sepsis, time spent in the emergency department, non-burn center ICU days, and hospital days were not significantly associated with six-month mortality in this cohort (all *p* > 0.05).

Although the odds ratios for coagulopathy and CVVHDF were large, their confidence intervals were wide, suggesting that these associations should be interpreted with caution, given the limited sample size and sparse events. The consistent direction of effect, however, aligns with their known impact on burn mortality reported in prior studies.

### 3.4. Multivariate Analysis

Logistic regression and Cox proportional hazards models were fitted to assess independent associations with six-month mortality, with TBSA and AKI included due to their strong univariate associations (*p* = 0.002), clinical relevance, low collinearity, best fit, and model stability (Table 3 and Table 4).

Alternative models were tested with variable results, but the small number of events prevented a more complete multivariable exploration, limiting the ability to confirm independence of any other variables.

In the multivariate logistic regression model, TBSA was significantly associated with increased mortality odds (OR = 1.09, 95% CI [1.03–1.17], *p* = 0.008), indicating an 9% increase in odds per 1% TBSA increase. AKI was also a strong predictor (OR = 9.67, 95% CI [1.78–79.41], *p* = 0.015), suggesting patients with AKI had 9.7 times higher odds of mortality compared to those without, after adjusting for TBSA. The overall model fit was good (AIC = 39.4).

In the Cox model, with survival time censored at 180 days, TBSA significantly increased the hazard of mortality (HR = 1.05, 95% CI [1.02–1.08], *p* < 0.001), with a 5% increase in hazard per 1% TBSA increase. Patients who developed AKI (HR = 3.48, 95% CI [1.18–10.29], *p* = 0.024) had a 3.5-fold higher risk of death over time compared with those without AKI. The model demonstrated strong predictive ability (concordance = 0.82, SE = 0.05; likelihood ratio test *p* = 7 × 10^−6^).

Together, both multivariate approaches demonstrated that TBSA and the occurrence of AKI showed consistent associations with mortality in this cohort, after adjusted analyses. The Cox model additionally showed that these effects persisted throughout the six-month follow-up period.

### 3.5. Reasons for Transfer

**Figure 3:**
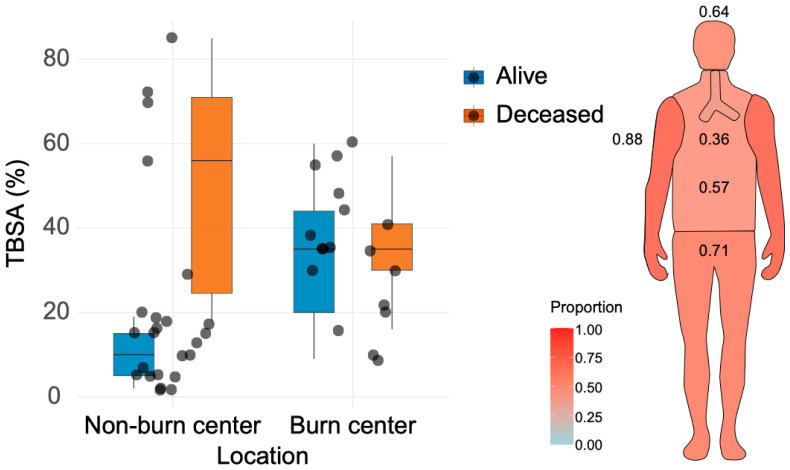
Proportional distribution of burned body regions in the overall cohort and assessment of TBSA according to treatment location and status at six months.

To assess whether patients with higher TBSA were transferred to burn centers, TBSA was compared between patients treated in the non-burn center (n = 24) and those transferred to a burn center (n = 18). The extent of TBSA burned differed significantly according to treatment location. Patients transferred to a burn center had larger burns than those treated in the non-burn center, with a median TBSA of 35% (IQR 19.2) compared to 15% (IQR 14.2), respectively (*p* = 0.0036, Wilcoxon rank-sum test), reflecting severity-driven referral decisions consistent with burn guidelines. However, several high-TBSA patients were not transferred due to additional factors like patient instability or burn-bed availability, highlighting implementation constraints. When stratified by survival status, among survivors, transferred patients also had a significantly greater burn extent (35% [IQR 24] vs. 10% [IQR 10], *p* = 0.0014). However, among non-survivors, there was no statistically significant difference between those treated in the non-burn center and those transferred (56% [IQR 46.5] vs. 35% [IQR 11], *p* = 0.458) (Figure 3).

Regarding the proportional distribution of burned body regions, most burns were located at the level of the extremities, followed by the head and torso. Inhalation injury was found in one-third of cases (Figure 3).

**Figure 4:**
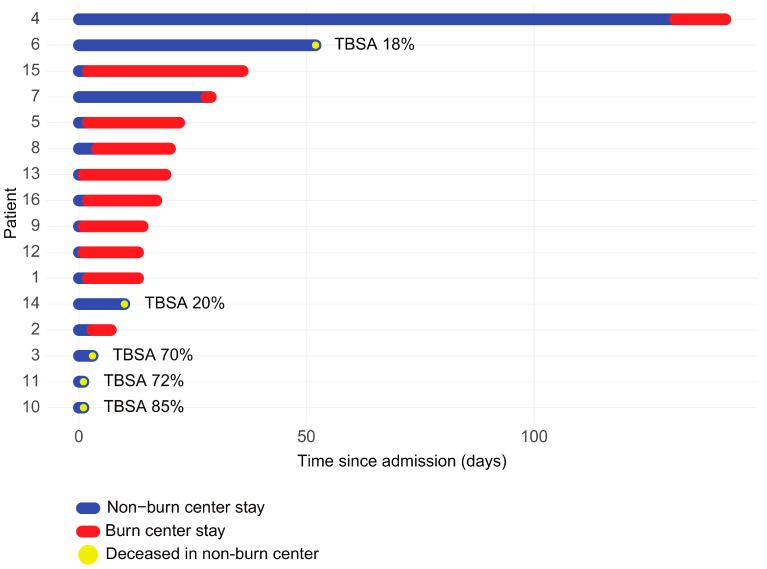
Individual patient trajectories showing days spent in non-burn (blue) and burn centers (red) from admission to death (deceased patients only). Patient 6 was 81 years old with a CCI of 4, and patient 14 was a 46-year-old woman who developed septic shock due to MRSA, leading to multiple organ failure.

The timeline visualization (Figure 4) shows the individual patient trajectories from admission to the non-burn center ICU to death, distinguishing the duration of hospitalization in the non-burn center (blue) and burn center (red). Patients who died without transfer (blue only) generally had shorter survival times and were often those with severe burns or early multi-organ failure, precluding safe transfer. By contrast, those who were transferred to a burn center typically survived longer post-admission, suggesting initial successful stabilization and triage, although mortality still occurred later in the course.

## 4. Discussion

### 4.1. Perspective for Clinical and Assistive Practice

From a broader scientific perspective, the advancement of burn care requires a multidisciplinary, evidence-based approach to optimize and ensure better clinical outcomes. Treating burn patients in non-burn centers does not always ensure the optimal or desirable quality of care due to limitations in infrastructure, logistics, and local expertise. Recent literature data emphasize the essential role of multidisciplinary teams (surgeons, intensivists, nurses, and psychosocial and rehabilitation needs) in addressing the acute and stabilization phases, which occur in non-burn settings, where resources and expertise are often lacking [9,25]. Furthermore, survival data provided by these non-specialized burn hospitals remain limited [26].

This cohort study addresses this gap by reporting the overall survival of all adult burn patients admitted initially to an ICU of a non-burn center and identifying independent mortality predictors in a non-burn center setting, a setting underrepresented in burn care research. The emphasis on “initially admitted” highlights that all patients shared the same entry point in our non-burn center ICU, regardless of the subsequent pathway. For non-transferred patients, the initial ICU phase was followed by a ward phase within the same hospital.

Establishing safe and effective clinical guidelines for burn management in non-burn centers is essential to improving patient outcomes.

With regard to our primary objective, in this study of 42 adult burn patients admitted initially to the ICU of a non-burn center, the overall six-month survival rate was 62%. The majority of deaths occurred within the first 60 days after injury, a period characterized by shock development, increased infection risk, and multi-organ failure. There is a subsequent plateau phase of the survival curve, which suggests that patients who survived the initial critical phase generally have favorable midterm outcomes.

In this cohort, aside from increased TBSA, patients with higher CCI scores, AKI, hemodynamic instability, coagulopathy, or the need for CVVHDF had significantly worse survival. These findings reflect the combined impact of pre-existing comorbidities, systemic hypoperfusion, and multi-organ dysfunction on post-burn outcomes [22].

Concerning the secondary objective, both the extent of burn injury (TBSA) and the presence of AKI showed consistent independent associations with mortality in adjusted models. Our findings reinforce that even small incremental increases in burn size significantly worsen outcomes; each 1% increase in TBSA raised the odds of death by 9% (OR = 1.09, 95% CI [1.03–1.17], *p* = 0.008) and the hazard of death over time by 5% (HR = 1.05, 95% CI [1.02–1.08], *p* < 0.001). AKI was another independent prognostic factor, conferring nearly a tenfold higher odds and a threefold higher hazard of death in multivariate models. Importantly, AKI remained independently associated with death after adjustment for TBSA, underscoring its role as a marker of critical illness severity rather than simply a consequence of burn size. These results align with existing burn literature but are notable for documenting these associations in a non-burn center ICU setting, where resource constraints and limited burn expertise may further challenge optimal care delivery [24,27,28].

The identification of TBSA and AKI as independent predictors in this cohort underscores the importance of integrating these results into clinical and assistive practices. During the acute phase, accurate TBSA assessment and vigilant physician- and nurse-led fluid monitoring, along with early surgical intervention and graft management, are essential in non-specialized burn care settings. A TBSA ≥ 20% was associated with significantly worse survival (*p* < 0.001), consistent with prior evidence of a critical threshold around 40–45% [29,30]. AKI’s strong association with mortality highlights the importance of renal function monitoring and timely management, particularly in facilities lacking advanced renal support like continuous veno-venous hemodiafiltration (CVVHDF). Although the need for CVVHDF was linked to mortality in this study, its use in only five patients suggests it reflects critical illness severity rather than a direct causal effect.

As for the assessment of local referral practice, severe burns were associated with increased mortality across both treatment locations, with the highest median TBSA (56% [IQR 46.5]) in deceased patients treated in the non-burn center. Even though patients with more severe burns were more likely to be transferred, some patients with high TBSA were managed locally due to severe organ failure at admission and impossibility of transfer, or even due to transfer delays (lack of free beds in burn centers around the country). This finding, while consistent with guidelines where TBSA drives referral, suggests implementation challenges in Romania. Patients transferred to a burn center generally sustained more extensive burns (median TBSA 35%, IQR 19.2) compared to cases managed in the non-burn center (median TBSA 15%, IQR 14.2), and during the subacute phase, transfer decisions were likely based on burn severity rather than survival outcome.

We should highlight that the lack of observed differences between transferred and non-transferred patients does not diminish the role of burn centers. We have highlighted two cases that were treated in the non-burn center (TBSA 18% and 20%, respectively) and, unfortunately, resulted in death without transfer. For example, one was an 81-year-old male with a CCI of 4, and the other was a 46-year-old woman who developed septic shock due to MRSA, leading to multiple organ failure. The lack of infrastructure for burn management in non-burn centers may contribute to increased mortality.

Regarding the rehabilitation phase, among survivors, an increase in TBSA was generally associated with longer hospital stays, particularly for patients treated in the non-burn center. In contrast, deceased patients tended to have higher TBSA values but shorter hospital stays, reflecting the early mortality associated with extensive burns. Our hospital is not suitable for the rehabilitation phase due to limited dedicated staff, including physiotherapists, occupational therapists, and nurses. We do not have the infrastructure required to support long-term functional rehabilitation, like facilities for mobility training, psychosocial support, and scar management.

The trajectory analysis highlights the heterogeneity in the clinical course of fatal burn cases, suggesting two distinct mortality patterns: first, there is the early mortality within the non-burn center, and second, delayed mortality after transfer. These findings emphasize the critical importance of early resuscitation, organ support, and timely transfer decisions.

### 4.2. Limitations

The exploratory findings of this study highlight the potential for non-burn centers to manage initial severe burns, provided stabilization is robust, but the study has several limitations that require mentioning. First, the small sample size (42 patients, 16 deaths) restricted statistical power, widened confidence intervals, and limited the number of predictors that could be included in multivariate models. Second, the retrospective single-center design limits generalizability to other non-burn center ICUs. Finally, although we had follow-up survival data for patients transferred to burn centers, we lacked information on their management within those centers, which prevented a meaningful comparison of other clinical outcomes between treatment locations.

The designation of TBSA and AKI as independent predictors of mortality in this study is based on selective modeling due to low events. Other variables are not to be cast aside and could retain their independence in larger studies.

Despite these constraints, the strengths of this study lie in its insights into the overall survival of adult burn patients admitted initially to a non-burn center ICU (an underrepresented population in the literature). The identification of independent predictors of six-month mortality from the perspective of a resource-limited burn ICU adds to the understanding of risk stratification. Additionally, the extended follow-up period strengthens the assessment of medium-term survival.

The limitations mentioned in this study underscore the need for future larger, multicenter studies in these non-burn ICU settings to validate these findings. Exploration of additional predictors like hemodynamic instability, sepsis, transfusion requirement, rhabdomyolysis, and non-invasive ventilation is urgently needed.

This study provides rare data from a non-burn ICU, documenting predictors of mortality in a setting that reflects the reality of many worldwide healthcare systems, such as Romania’s, where burn patients are often managed outside specialized centers.

The care of patients who remain in non-burn centers extends into the early rehabilitation phase, where multidisciplinary teams manage infections, support nutrition and mobilization, and perform surgical debridement and autologous grafting, procedures that carry additional risks when such treatment options are not available in these facilities [25,31].

Reallocating resources, such as through telemedicine support from burn specialists or standardized training programs, could enhance quality of care [32]. Development of AI-based decision support tools integrating key clinical predictors may assist in early triage and management. On a broader scale, national burn societies could play a critical role by promoting standardized guidelines, training programs, telemedicine support, and improved triage coordination to optimize outcomes for burn patients managed in non-specialized facilities.

## 5. Conclusions

In this retrospective cohort of 42 adult burn patients initially admitted to a single Romanian university hospital non-burn ICU (average 7 patients/year), six-month survival was 62%, with TBSA and AKI independently associated with mortality after adjusted analyses. Death clustered early, reflecting the acute vulnerabilities of resource-limited hospitals. Though the small sample size limits definitive conclusions, these findings highlight the importance of a phase-specific, multidisciplinary approach to bolster clinical and assistive practices. Early identification of high-risk patients, structured monitoring of renal and hemodynamic function, coordinated nursing care, and timely transfer pathways may help improve outcomes across the early phases of burn management.

While transfer to specialized burn centers remains essential for patients with extensive injuries (TBSA > 20%), optimizing the initial stabilization and critical care provided in non-burn ICUs is equally important. Larger, multicenter non-burn hospital studies are needed to validate these results and guide the development of standardized protocols, improved triage systems, and supportive tools such as telemedicine and AI-based risk models to optimize outcomes in non-burn hospitals.

## Figures and Tables

**Table 1:** Patient baseline characteristics with burn data.

			Status at Six Months		
Variable ^1^	N ^1^	Overall ^1^ N = 42 ^1^	Alive n = 26 ^1^ (61.9%)	Dead n = 16 ^1^ (38.1%)	*p*-Value ^1^
**Patient demographics**					
***Age (years)***	42	57 (18)	51 (17)	68 (15)	**0.003**
***Gender***	42				0.7
F		11/42 (26%)	6/26 (23%)	5/16 (31%)	
M		31/42 (74%)	20/26 (77%)	11/16 (69%)	
***Charlson Comorbidity Index***	42				**<0.001**
0		16/42 (38%)	14/26 (54%)	2/16 (13%)	
1		4/42 (9.5%)	3/26 (12%)	1/16 (6.3%)	
2		4/42 (9.5%)	3/26 (12%)	1/16 (6.3%)	
3		5/42 (12%)	2/26 (7.7%)	3/16 (19%)	
4		7/42 (17%)	3/26 (12%)	4/16 (25%)	
5		1/42 (2.4%)	1/26 (3.8%)	0/16 (0%)	
6		4/42 (9.5%)	0/26 (0%)	4/16 (25%)	
8		1/42 (2.4%)	0/26 (0%)	1/16 (6.3%)	
**Clinical outcomes**					
***Treatment location***	42				0.2
Transferred to the burn center		18/42 (43%)	9/26 (35%)	9/16 (56%)	
Treated in the non-burn center		24/42 (57%)	17/26 (65%)	7/16 (44%)	
***Emergency room length of stay (minutes)***	42	148 (75)	149 (86)	148 (70)	0.7
***Non-burn center hospital length of stay (days)***	42	5 (16)	10 (22)	2 (5)	0.10
***Non-burn center ICU length of stay (days)***	42	3 (3)	3 (3)	2 (3)	0.8
***Time to burn center transfer (days)***	18	2 (1)	1 (1) n = 9	2 (1) n = 9	0.6
**Burn characteristics**					
***TBSA (%)***	42	20 (27)	14 (14)	37 (29)	**<0.001**
***Mechanism of burn***	42				0.8
Chemical burn		1/42 (2.4%)	1/26 (3.8%)	0/16 (0%)	
Electrical burn		8/42 (19%)	6/26 (23%)	2/16 (13%)	
Flame burn		29/42 (69%)	17/26 (65%)	12/16 (75%)	
Scald burn		4/42 (9.5%)	2/26 (7.7%)	2/16 (13%)	
***Circumstances***	42				0.8
Accidental (home, work, fishing)		39/42 (93%)	24/26 (92%)	15/16 (94%)	
Suicide attempt		1/42 (2.4%)	1/26 (3.8%)	0/16 (0%)	
**R-Baux Score**	42	83 (75, 106)	78 (69, 86)	110 (97, 125)	**<0.001**
**ICU physiological status**					
***Neurologic—on admission***	42				0.2
conscious		16/42 (38%)	12/26 (46%)	4/16 (25%)	
sedated		26/42 (62%)	14/26 (54%)	12/16 (75%)	
***Airway—on admission***	42				0.2
spontaneous breathing (+/− oxygen)		19/42 (45%)	14/26 (54%)	5/16 (31%)	
intubated and mechanically ventilated		23/42 (55%)	12/26 (46%)	11/16 (69%)	
***Hemodynamic—on admission***	42				**<0.001**
stable		25/42 (60%)	21/26 (81%)	4/16 (25%)	
requiring vasopressor		17/42 (40%)	5/26 (19%)	12/16 (75%)	
***Renal—on admission***	42				**0.009**
>0.5 mL/kg		29/42 (69%)	22/26 (85%)	7/16 (44%)	
oliguria		11/42 (26%)	4/26 (15%)	7/16 (44%)	
anuria		2/42 (4.8%)	0/26 (0%)	2/16 (13%)	
**Complications**					
***ARDS***	42	5/42 (12%)	2/26 (7.7%)	3/16 (19%)	0.4
***AKI***	42	16/42 (38%)	5/26 (19%)	11/16 (69%)	**0.004**
***Coagulopathy***	42	9/42 (21%)	1/26 (3.8%)	8/16 (50%)	**<0.001**
***Rhabdomyolysis***	42	25/42 (60%)	14/26 (54%)	11/16 (69%)	0.5
***Sepsis***	42	13/42 (31%)	9/26 (35%)	4/16 (25%)	0.7
***Transfusion***	42	13/42 (31%)	6/26 (23%)	7/16 (44%)	0.2
***CVVHDF***	42	5/42 (12%)	0/26 (0%)	5/16 (33%)	**0.004**

^1^ Mean (SD) for normally distributed variables (age); median (IQR) for non-normally distributed variables, n/N (%) for categorical variables. ICU—intensive care unit; TBSA—total body surface area; R-Baux Score—Revised Baux Score; ARDS—acute respiratory distress syndrome; CVVHDF—continuous venous–venous hemodiafiltration; AKI—acute kidney injury. *p* < 0.05: statistically significant difference between alive and deceased groups.

**Table 2:** Univariate logistic regression analysis of predictors of six-month mortality.

Variable	Contrast	Odds Ratio (95% CI)	*p*-Value
Age (years)	per 1-year increase	1.06 (1.02–1.12)	0.007
TBSA (%)	per 1% increase	1.08 (1.03–1.13)	0.002
Charlson Comorbidity Index (>2 vs. ≤2)	binary	10.0 (2.52–48.1)	0.002
AKI (Yes vs. No)	binary	9.24 (2.34–43.1)	0.002
Coagulopathy (Yes vs. No)	binary	25.0 (3.79–502.4)	0.005
Hemodynamic Instability (Unstable vs. Stable)	binary	12.0 (2.8–68.6)	<0.001
CVVHDF (Yes vs. No)	binary (Firth correction)	25.3 (2.54–3432.9)	0.003
Treatment Location (Transferred vs. Treated locally)	binary	2.43 (0.69–9.04)	0.17
Sepsis (Yes vs. No)	binary	0.63 (0.14–2.44)	0.51
Time Spent in ER (min)	continuous	1.00 (0.99–1.01)	0.74
Non-burn center ICU days	continuous	1.03 (0.97–1.11)	0.34
Non-burn center hospital days	continuous	1.00 (0.97–1.03)	0.99

Odds ratios (ORs) and 95% confidence intervals (CIs) are derived from univariate logistic regression models using the Wald test. CVVHDF was analyzed with Firth’s penalized logistic regression due to complete separation. *p*-values < 0.05 indicate statistical significance.

**Table 3:** Multivariate logistic regression assessing associations with six-month mortality in burn patients admitted initially to a non-burn center ICU.

Variable	Adjusted OR (95% CI)	*p*-Value
TBSA (%)	1.09 (1.03–1.17)	0.008
Acute Kidney Injury (AKI)	9.67 (1.78–79.41)	0.015

Residual deviance = 33.4 (vs. 55.8 null); AIC = 39.4. Both TBSA and AKI remained independently associated with six-month mortality.

**Table 4:** Cox proportional hazards regression evaluating independent predictors of time-to-death. Hazard ratios (HRs) with 95% confidence intervals.

Variable	Adjusted HR (95% CI)	*p*-Value
TBSA (%)	1.05 (1.02–1.08)	<0.001
Acute Kidney Injury (AKI)	3.48 (1.18–10.29)	0.024

N = 42; events = 16; concordance = 0.82 (SE = 0.05); likelihood ratio χ^2^ = 23.7 (df = 2, *p* = 7 × 10^−6^).

## Data Availability

The data that support the findings of this study are available from the corresponding author, C.C., upon reasonable request. Figure 3 was partly generated using Servier Medical Art, provided by Servier, licensed under a Creative Commons Attribution 3.0 Unported License.

## Supplementary Materials

The following supporting information can be downloaded at: https://www.mdpi.com/article/10.3390/medicina62030443/s1. Supplementary Figure S1. Flow diagram illustrating the selection process of the study cohort. A total of 45 patients with burn injuries identified between 1 January 2019 and 31 December 2024, were assessed for eligibility. Three patients were excluded for not meeting the inclusion criteria, resulting in 42 patients included in the final analysis. Supplementary File S1. Completed STROBE checklist for this cohort study.
