# Supplementary figures and images for: Outcomes of Burn Patients Admitted Initially to a Non-Burn Center Intensive Care Unit in Romania: A Retrospective Exploratory Study
Source: Medicina (Kaunas). 2026 Feb 26;62(3):443. doi: 10.3390/medicina62030443 (PMC13028255; doi:10.3390/medicina62030443)

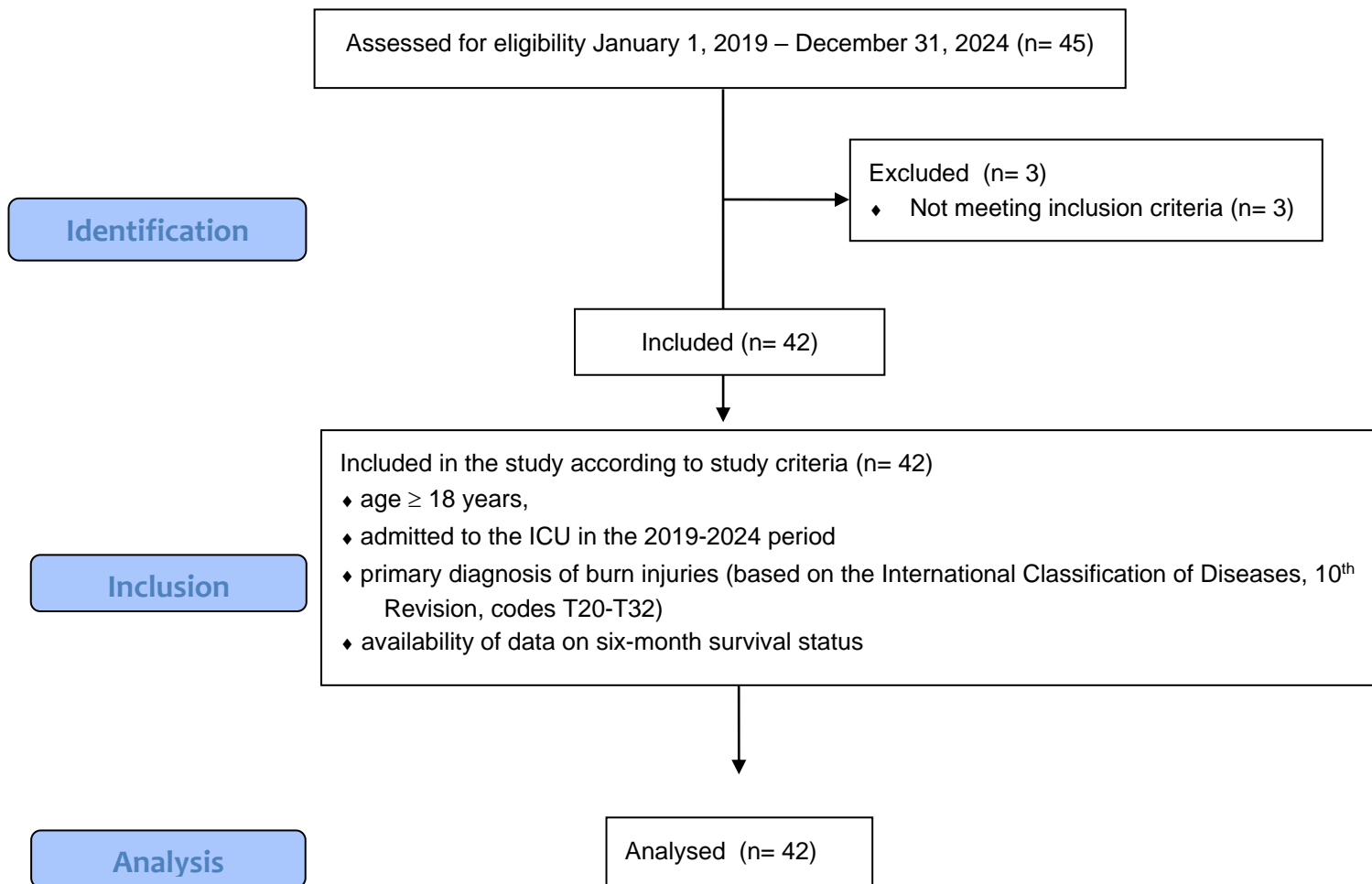

Supplement: Supplementary file 1 [file medicina-62-00443-s001.zip › Supplementary Figure S1.pdf]
